# State of development of regional hospice and palliative care networks in Germany and a descriptive comparison of the role of coordination: results of an online survey using a maturity model

**DOI:** 10.1186/s12904-026-02279-7

**Published:** 2026-08-14

**Authors:** Hanna A.A. Röwer, Eileen Doctor, Christoph Buck, Franziska A. Herbst, Sven Schwabe

**Affiliations:** 1 Institute for General Practice and Palliative Care, Hannover Medical School, Carl-Neuberg-Straße 1, Hannover, 30625 Germany; 2 Research Institute for Information Management, University of Applied Sciences Augsburg, Alter Postweg 101, Augsburg, 86159 Germany; 3 Centre for Future Enterprise, School of Management, Queensland University of Technology, 2 George Street, Brisbane, QLD 4000 Australia

**Keywords:** Community networks, Palliative care, Health personnel, Intersectoral collaboration

## Abstract

**Background:**

Regional hospice and palliative care networks (RHPCNs) aim to establish frameworks for care-management level collaboration between end-of-life care providers within specific regions. Since 2022, political support for RHPCNs in Germany has been bolstered by a new funding guideline for network coordination under § 39d SGB V. However, despite this political backing, limited data exist on the development of these networks. The present study, conducted within the framework of the HOPAN project, aimed to systematically assess the maturity of RHPCNs in Germany and offer first insights in differences in observed maturity levels, further depending on coordination.

**Methods:**

A maturity model comprising six dimensions (moderation, infrastructure, public relations and information exchange, establishment and development, training and educational programs, development of regional care services and practices), further divided into *n* = 28 subdimensions developed de novo for the RHPCN context was applied as a self-assessment instrument. An online survey was conducted between November 2023 and January 2024. Invitations were sent via e-mail to *N* = 390 contacts involved in RHPCNs and disseminated through newsletters of two professional associations. Data were descriptively analyzed using SPSS.

**Results:**

A total of *n* = 70 complete datasets were collected, *n* = 64 being included in the final analysis. The analyzed RHPCNs were established between 2001 and 2024. The “Financing” subdimension received the least mature self-assessments, with 46.7% of participating RHPCNs (*n* = 28) self-classifying at the lowest level. The “Structure of network meetings” subdimension demonstrated the highest level of maturity, with 42.6% of RHPCNs (*n* = 26) self-classifying full maturity. A comparative overview of maturity based on coordination type revealed that RHPCNs with paid coordination tended to exhibit more structured organization, systematic communication and funding, and comprehensive planning; moreover, also volunteer coordinators and coordination by network members demonstrated greater maturity than RHPCNs with no coordination at all.

**Conclusion:**

While many RHPCNs have well-structured coordination practices and effective meeting formats, significant challenges remain concerning the procurement of stable and systematic funding and project management. These insights suggest a need for targeted strategies to improve infrastructure and support the establishment of effective coordination roles to encourage the further development and sustainability of RHPCNs.

**Trial registration:**

The study was prospectively registered in the German Clinical Trials Register (Deutsches Register Klinischer Studien Registration N° DRKS00030629 date of registration November 2, 2022). The study is searchable under the International Clinical Trials Registry Platform Search Portal of the World Health Organization under the same registration number. Ethical approval was granted by the Ethics Committee of Hannover Medical School on July 20, 2022 (N° 10424_BO_S_2022). Participants provided informed consent on the first page of the survey, agreeing to the processing of their data in accordance with the ethics committee approval. Participation was contingent on this consent.

**Supplementary Information:**

The online version contains supplementary material available at 10.1186/s12904-026-02279-7.

## Introduction

In palliative care, multiprofessional and interdisciplinary cooperation among healthcare providers promotes the delivery of comprehensive and high-quality care to patients and families confronting life-threatening illnesses [1–4]. In this context, regional hospice and palliative care networks (RHPCNs) represent a relatively recent innovation, promoting communication and collaboration between diverse healthcare organizations, enabling patient access to appropriate services, ensuring continuity of care, and strengthening the competencies of healthcare providers [5–12]. RHPCNs play a key role in the organization of quality circles and training sessions, the development of regional standards and public awareness campaigns, and the maintenance of low-threshold and consistent contact between outpatient and inpatient service providers across disciplines [6, 7, 12–14]. Through these actions, they have the potential to improve the quality of palliative care and enhance patient satisfaction at a regional level [5–15].

In recent years, there has been a notable expansion in the number of RHPCNS in Germany [16], likely driven by the introduction of a new funding guideline under § 39d of the German Social Security Code, Fifth Book (SGB V), in 2022 [17]. This regulation allows for the funding of one RHPCN per municipality or district, with health insurance funds providing up to EUR 15,000, contingent upon matching financial contributions from the municipality. The primary objective of this policy is to strengthen infrastructure and foster collaboration by enabling the employment of a paid coordinator, thereby enhancing regional cooperation among healthcare providers to better support individuals at the end of life [18]. Preliminary insights from general healthcare networks and international examples underscore the value of coordination in improving clinical quality, health outcomes, and patient satisfaction, while simultaneously reducing healthcare costs [19]. For example, in the Netherlands, RHPCNs, often spearheaded by coordinators, have been shown to facilitate collaboration among healthcare providers, improve service accessibility, and increase quality of care [20].

While the introduction of funding for these RHPCNs in Germany represents a significant advancement, it also highlights the lack of standardized quality criteria and reliable evidence regarding the effectiveness of RHPCNs and the impact of their structural characteristics (e.g., coordination). Moreover, the absence of benchmarks and established performance metrics makes it challenging to systematically assess the impact of these RHPCNs and draw meaningful comparisons between regions.

To address this deficit of information regarding the status of RHPCNs in Germany, the HOPAN project (Funding: Innovation Fund of the German Federal Joint Committee (G-BA), Grant N° 01VSF22042) implemented a maturity model as an initial framework to assess and further derive recommendations to enhance the operational maturity of RHPCNs.

Maturity models represent valuable tools for the assessment and improvement of organizational capabilities [21, 22]. By defining various levels of efficiency, manageability, and measurement, these models are able to assess organizational status, identify challenges, and implement targeted improvements [23, 24]. Originating primarily from the fields of software development and engineering, maturity models have increasingly been adapted for use in the healthcare sector [25], primarily to support hospitals in managing digital transformation challenges (e.g., telemedicine) and analyze care pathways [24]. In this context, they may contribute to improving healthcare practices by identifying tensions between practical implementation (of, e.g., innovative solutions such as digital health technologies and telemedicine) and research [26], while also facilitating organizational management and enhancing service quality [27, 28].

The HOPAN maturity model aims at establishing clear evaluation criteria for RHPCNs, enabling stakeholders to systematically measure progress. In more detail, the initial maturity model assesses RHPCNs’ capacity to establish efficient relationships, with a focus on strategy and organizational structure.

### Study aim

The present study examined the current state of development of RHPCNs across Germany, identifying areas in which participating RHPCNs were more advanced or less mature. The identification of less mature fields aimed at facilitating the identification of areas in need of improvement. Moreover, the study sought to describe the differences in observed maturity levels depending on the presence of coordination and utilization of the new funding under § 39d SGB V.

## Methods

### Design

The maturity model applied in the present study was developed in the HOPAN research project and is documented in a prior publication [29]. For the model development, an existing maturity model for healthcare networks [30], comprising four dimensions, was adapted for the RHPCN use case and expanded to six dimensions: dimensions and subdimensions of the maturity model for RHPCN were derived through focus group interviews with *n* = 19 network coordinators and other experts involved in the management and establishment of RHPCNs during *n* = 6 online workshops held between May and August 2023 using a structured consensus process. The model comprised six dimensions, each of which contained a varying number of subdimensions (see Table 1). Each subdimension comprised four levels, corresponding to four development stages: initial/ad hoc (level 0), structured (level 1), established (level 2), and optimized (level 3).

The model was subsequently adapted into an online questionnaire, with the definitions of individual levels serving as the response options for each subdimension. The questionnaire was supplemented by structural questions about the RHPCNs and sociodemographic items. The final questionnaire included 103 items (see appendix), of which 28 addressed the maturity model classification; 63 dealt with network structure and 12 were open questions exploring networking challenges and best practices, which were analyzed and presented separately [31, 32].

**Table 1:** Maturity model with dimension descriptions, as presented in the survey

Dimension with definition	Subdimensions
Moderation Network moderation serves a central, neutral, steering role at an operational level, typically carried out by a head office. Depending on the network’s legal form, this role may be assumed by coordinators. Responsibilities include the coordination of network communication and cooperation, needs assessment and agenda setting, the structuring and organization of network meetings, and the management of information and process flows.	• Role of coordination (nature of coordination) • Role of network members • Definition of topics • Structure of network meetings • Organization of network meetings • Exchange of information
Establishment and development Establishment and development supports network growth through the identification and admission of new members, the analysis of objectives and needs, and the determination of regional network activities and stakeholder engagement.	• Identification and approach of network members • Enrollment of network members • Needs identification of (potential) network members • Objectives • Target area: structure/region
Infrastructure Network infrastructure encompasses all measures needed for the physical and informal operation of the network, including communication structure, financing, IT infrastructure, and legal form. These components serve as the foundation for all network activities.	• Communication: data storage and information dissemination • Financing • Legal form of organization
Public relations and information exchange Public relations ensures the network is perceived as credible and established by the public and, above all, target groups. It involves understanding the target group’s needs, developing information materials, disseminating content, and collaborating with external bodies.	• Target group focus • Development of joint materials • Design and corporate identity • Dissemination of information/public relations channels • Scope and effectiveness of public relations work • Cooperation with external bodies (e.g., regional support centers, information centers, professional associations, higher-level networks)
Training and educational programs Training and educational programs enhance members’ qualifications through training and advanced education. This dimension involves the identification of training needs, the organization and funding of courses, and the evaluation of their effectiveness and quality.	• Identification of training and advanced education needs • Organization of training and advanced education programs • Financing of training and advanced education programs • Evaluation of further and advanced education programs
Development of regional care services and practices Further development of regional care services and practices aims to improve local care by identifying, developing, implementing, and evaluating joint projects to better meet community needs. In this process, network members are actively engaged.	• Identification of joint projects (e.g., brochures, regional care standards, information events) • Development and implementation of new joint projects • Evaluation and implementation of new joint projects • Involvement of network members in the development of new joint projects

### Data collection

The maturity model was translated into an online questionnaire using SoSciSurvey (Version 3.5.02, Munich, Germany), together with structural questions compiled on the basis of a systematic literature review [16]. The survey was piloted in November 2023 with a sample size of *n* = 5 network coordinators. Pilot participants were systematically selected from those involved in the development workshops during the preceding project phase. Network coordinators taking part in the pretest were selected in accordance with their varying levels of experience with scientific work, participation in online surveys, and network coordination experience. The pretest focused on the clarity of the categorization instructions and the adequacy of the answer options for the supplementary structural questions. For this purpose, feedback was collected via open text entry for each question.

Following survey refinement on the basis of this feedback, the final version was made available online for 57 days (November 29, 2023 to January 24, 2024), incorporating a 10-day extension of the originally planned data collection period. The first page of the survey presented information about the study purpose and data protection measures, and participants could only proceed after providing informed consent.

### Recruitment

The recruitment process utilized a comprehensive contact database of RHPCNs throughout Germany. This database, developed during a preceding project phase aimed at identifying the number of German RHPCNs [16], was subsequently updated to include recently established RHPCNs. In addition, contact information for the individuals responsible for the governance or oversight of these RHPCNs was included to facilitate the dissemination of invitations. Based on this updated contact list, study invitations were sent to *N* = 390 contacts. Additionally, the survey was announced in the newsletters of two major associations for palliative care and hospice services in Germany: the German Association for Palliative Medicine (“Deutsche Gesellschaft für Palliativmedizin”) and the German Hospice and Palliative Association (“Deutscher Hospiz- und PalliativVerband”).

Throughout the survey period, three reminder emails were sent. The first reminder was sent to all original contacts, and two additional reminders were sent to those identified by the survey system as having initiated but not yet completed the survey.

### Data analysis

Following the survey administration, the collected data were subjected to a series of rigorous quality checks to ensure compliance with the predefined inclusion and exclusion criteria. The exclusion criteria included: (1) surveys terminated prematurely, prior to completion of the final page; (2) networks exclusively providing multiprofessional care without engaging in structural activities (e.g., case management); (3) supraregional structures, including national and regional associations; and (4) networks operating exclusively within the field of pediatric care. Duplicate responses were identified through an analysis of network details and manually excluded.

Data from interprofessional care structures, such as specialized palliative home care teams (German abbreviation: SAPV), were included only when the structures were also active at the level of care management (as opposed to pure case management). Care management was defined as encompassing the organization of regular meetings with cooperating partners, the ongoing development of regional structures, and the provision of additional training for network members. Adherence to the criteria was ascertained through an analysis of the questionnaire data and further research on the websites of any RHPCNs that voluntarily disclosed their network names (i.e., those seeking individual results). For the data analysis, network names were excluded from the dataset.

Data were analyzed using IBM SPSS Statistics Version 28 (SPSS Inc., Chicago, IL, USA). Classification of maturity level (ranging from 0 to 3) was evaluated descriptively, with corresponding values presented. Each subdimension was assigned a minimum value of 0 and a maximum value of 3. The distribution of RHPCNs across maturity levels was assessed both numerically and as a percentage. To enable comparison across networks with different coordination structures, results from the maturity-level assessment were additionally stratified by coordination type (no coordination, only voluntary coordination, at least one employed coordinator and some kind of coordination). As the underlying maturity-level data are ordinally scaled, the median was used as the appropriate measure of central tendency for these group comparisons.

In view of the extant literature, different types of coordination (i.e., voluntary coordination, at least one paid coordinator, no coordination, any coordination) were identified for inclusion in the subsequent analysis. To compare maturity levels across these coordination types, distribution between levels for the individual subdimensions were analyzed and presented in tabular form.

All subdimensions were optional; no item required a mandatory response. Varying sample sizes across subdimensions therefore reflect a structural feature of the survey design. The reasons for individual non-response to specific subdimensions cannot be determined from the available data.

## Results

The survey was accessed *N* = 408 times and completed *n* = 70 times. Incomplete data records were excluded from the analysis. Dropouts occurred predominantly in the early sections of the questionnaire, suggesting that incomplete records were unlikely to represent partially considered responses as the survey was also voluntary and self-administered. Only records in which participants had navigated to the final page of the survey were retained for analysis. Following further plausibility checks and data cleansing following the described exclusion criteria, 64 datasets were included in the final analysis.

### Sociodemographic data

Data from the *N* = 64 RHPCNs were provided by 65 participants (with one questionnaire completed collaboratively by two individuals). The largest group in terms of professional background comprised nursing and nursing science (*n* = 24; 37.5%) (see Table 2). Respondents were allowed to select multiple professional backgrounds (up to three). Regarding professional roles, the majority of respondents (*n* = 38; 59.4%) reported a coordinating position. Overall, 47.6% (*n* = 30) reported more than 15 years of experience in end-of-life care, whereas only 4.8% (*n* = 3) reported less than 1 year of experience. Detailed information on structure and coordinator profiles in German RHPCNs is available in a prior publication by the same author group [31].

**Table 2:** Sociodemographic data

			*N*	%
Gender	Total		65	
Female			46	70.8
Male			19	29.2
Other			0	0
Age		*Total*	65	
18–30			1	1.5
31–43			10	15.4
44–56			18	27.7
57–69			34	52.3
** ≥** 70			2	3.1
Highest educational qualification	*Total*		64	
Apprenticeship			15	23.4
Vocational school/advanced training program			5	7.8
Bachelor’s degree or intermediate diploma			6	9.4
Master’s degree or diploma			21	32.8
PhD			12	18.8
Other training (e.g., acquired abroad)			5	7.8
Professional background (multiple choice, percentage of cases)	*Total*		64	
Nursing/care management			24	37.5
Education/social work			15	23.4
Administration/economics/health management			12	18.8
Medicine			12	18.8
Social sciences/humanities/public health			6	9.4
Physiotherapy			1	1.6
Other			8	12.5
Role in RHPCN		*Total*	64	
Coordination			38	59.4
Steering group/executive board			12	18.8
Employee of network partner			4	6.3
Other			10	15.6
Job experience in the field of hospice/palliative care		*Total*	63	
< 1 year			3	4.8
1–3 years			7	11.1
3–7 years			9	14.3
7–15 years			14	22.2
> 15 years			30	47.6

### Network structural data

**Fig. 1:**
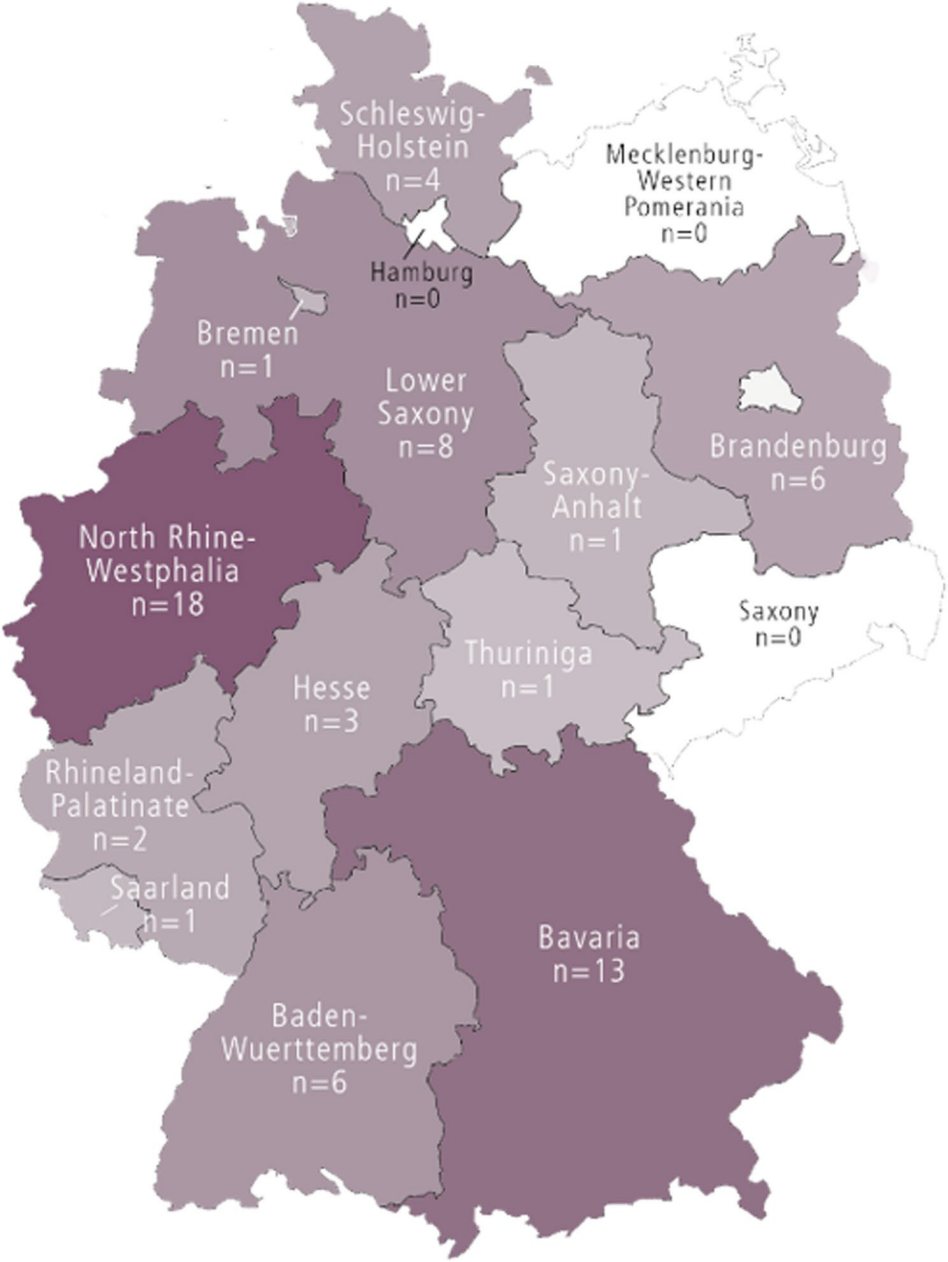
Participating RHPCNs by German federal state

**Fig. 2:**
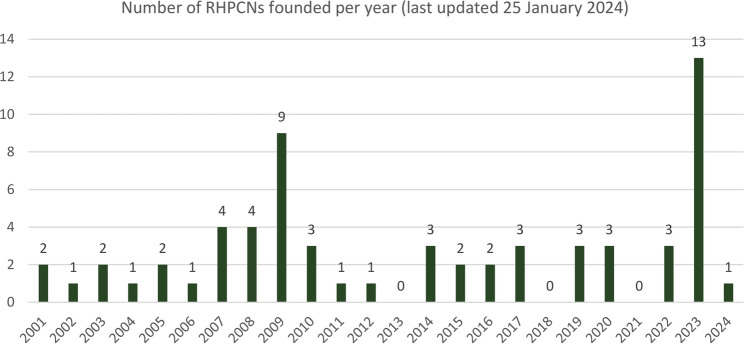
Number of RHPCNs founded per year

The majority of the participating RHPCNs (*N* = 64) were located in North Rhine–Westphalia (*n* = 18, 28.1%), followed by Bavaria (*n* = 13, 20.3%) and Lower Saxony (*n* = 8, 12.5%) (see Fig. 1). They were founded between 2001 and 2024, with most established in 2023 (*n* = 13, 20.3%) (see Fig. 2). Regarding geographical scope, most RHPCNs were active within a single administrative district (*n* = 36, 56.3%), followed by those operating beyond one administrative district (*n* = 17; 26.6%) and those covering only parts of one administrative district (*n* = 11; 17.2%). Further details on structural characteristics can be found in previous articles [16, 31].

### Moderation

The dimension of moderation comprised six subdimensions. The subdimension with the highest proportion of RHPCNs at the lowest maturity level (level 0) was “Definition of topics,” with 25.4% (*n* = 16) of RHPCNs at this level. This subdimension was characterized by spontaneous vs. structured and forward-looking topic setting aligned with common strategic goals (e.g., political framework conditions). Conversely, the subdimension with the greatest proportion of RHPCNs at the highest maturity level (level 3) was the “Structure of network meetings,” with 42.6% (*n* = 26) of RHPCNs self-assessing in this level. This subdimension described the preparedness and structure of meetings, encompassing agendas, topic prioritization, minute-taking, and systematic follow-up. Across the remaining subdimensions, the distribution of RHPCNs across maturity levels varied considerably. For “Role of coordination,” responses were relatively evenly spread, with the largest share of networks at level 2 (41.3%, *n* = 26), while levels 0, 1, and 3 each accounted for roughly one fifth of networks. “Role of network members” and “Organization of network meetings” were both concentrated at level 1. For “Exchange of information,” most RHPCNs fell within levels 1 and 2 (38.1% and 41.3% respectively), with relatively few networks at the highest level (14.3%, *n* = 9). Overall, the findings indicated that RHPCNs demonstrated relatively low maturity in the dimension of moderation (see Table 3).

**Table 3:** Network maturity regarding moderation

	*n*	Level 0	Level 1	Level 2	Level 3
Role of coordination (nature of coordination)	63	11 (17.5%)	13 (20.6%)	26 (41.3%)	13 (20.6%)
Role of network members	63	8 (12.7%)	29 (46.0%)	14 (22.2%)	12 (19.0%)
Definition of topics	63	16 (25.4%)	27 (42.9%)	9 (14.3%)	11 (17.5%)
Structure of network meetings	61	3 (4.9%)	10 (16.4%)	22 (36.1%)	26 (42.6%)
Organization of network meetings	61	5 (8.2%)	27 (44.3%)	11 (18.0%)	18 (29.5%)
Exchange of information	63	4 (6.3%)	24 (38.1%)	26 (41.3%)	9 (14.3%)

### Establishment and development

The dimension of establishment and development comprised five subdimensions. The subdimension with the highest proportion of RHPCNs at the lowest maturity level (level 0) was “Enrollment of network members,” with 13.0% (*n* = 9) of RHPCNs at this level. This subdimension reflected the procedures associated with the (formal) integration of new members into the RHPCNs. Interestingly, this subdimension also had the highest proportion of RHPCNs at the highest maturity level (level 3), with 30.4% (*n* = 21) of RHPCNs demonstrating well-established and standardized enrollment processes. The subdimension “Identification and approach of network members” showed a concentration of RHPCNs at level 2 (51.6%, *n* = 32), with 23.4% (*n* = 15) reaching the highest maturity level. “Needs identification of (potential) network members” was also a relatively low mature subdimension, with 56.5% (*n* = 39) of networks at level 1 and only 14.5% (*n* = 10) at level 3. “Objectives” and “Target area: structure/region” showed comparable distributions, with the majority of networks at levels 1 and 2 (70.6% and 67.6% respectively) and roughly one fifth reaching level 3. Overall, findings in this dimension suggest that while basic structures for member identification and network building are largely in place, more systematic and needs-oriented approaches to member engagement and enrollment remain an area for further development across the majority of RHPCNs (see Table 4).

**Table 4:** Network maturity regarding establishment and development

	*n*	Level 0	Level 1	Level 2	Level 3
Identification and approach of network members	63	8 (12.5%)	8 (12.5%)	32 (51.6%)	15 (23.4%)
Enrollment of network members	63	9 (13.0%)	31 (44.9%)	8 (11.6%)	21 (30.4%)
Needs identification of (potential) network members	63	1 (1.4%)	39 (56.5%)	19 (27.5%)	10 (14.5%)
Objectives	62	5 (7.4%)	21 (30.9%)	27 (39.7%)	15 (22.1%)
Target area: structure/region	63	4 (5.8%)	22 (31.9%)	25 (35.7%)	17 (26.1%)

### Infrastructure

The dimension of infrastructure comprised three subdimensions. The subdimension with the highest proportion of RHPCNs at the lowest level (level 0) was “Financing,” with 46.7% (*n* = 28) of RHPCNs at this level. This subdimension reflected the degree of systematic funding sources, with low levels indicating that financial resources were typically acquired on an ad hoc basis for specific activities, often without structured documentation. Conversely, the subdimension with the highest proportion of RHPCNs at the highest level (level 3) was “Legal form of organization,” with 27.6% (*n* = 16) of RHPCNs achieving this level. This subdimension pertained to the formal legal structure of the RHPCNs, with higher levels reflecting legal forms aligned with strategic goals and regular evaluation.

“Communication: data storage and information distribution” showed the most concentrated distribution, with 56.5% (*n* = 35) of networks at level 2, while comparatively few reached the highest maturity level (12.9%, *n* = 8). Overall, the findings showed that, while many RHPCNs demonstrated well-developed communication and legal structures, financial sustainability was a significant challenge (see Table 5).

**Table 5:** Network maturity regarding infrastructure

	*n*	Level 0	Level 1	Level 2	Level 3
Communication: data storage and information distribution	63	13 (21.0%)	6 (9.7%)	35 (56.5%)	8 (12.9%)
Financing	60	28 (46.7%)	14 (23.3%)	10 (16.7%)	8 (13.3%)
Legal form of organization	58	19 (32.8%)	6 (10.3%)	17 (29.3%)	16 (27.6%)

### Public relations and information exchange

The dimension of public relations and information exchange comprised six subdimensions. The subdimension with the highest proportion of RHPCNs at the lowest level (level 0) was “Design and corporate identity,” with 21.0% (*n* = 13) of RHPCNs at this level. This subdimension reflected the degree of adherence to corporate design guidelines, with respect to consistency and the updating of older materials to align with current corporate designs. Notably, “Design and corporate identity” simultaneously showed the most even distribution across all four maturity levels. Conversely, the subdimension with the greatest proportion of RHPCNs at the highest level (level 3) was “Cooperation with external bodies,” with 30.6% (*n* = 19) of RHPCNs achieving this level. This subdimension reflected the degree of structured and needs-oriented collaboration with external organizations, such as regional advice centers, professional associations, and broader RHPCNs. “Target group focus” showed the most concentrated distribution, with 90.3% of networks at levels 1 and 2 and only 4.8% (*n* = 3) reaching level 3. “Dissemination of information/public relation channels” and “Scope and effectiveness of public relations work” showed comparable patterns, with the majority of RHPCNs at level 1 and few reaching the highest maturity level (8.1% each). Overall, the findings indicated that while cooperation with external bodies and the development of a corporate identity appear comparatively advanced, the more active and strategic aspects of public relations work — including targeted dissemination and audience-specific communication — remain areas for further development across most participating RHPCNs (see Table 6).

**Table 6:** Network maturity regarding public relations and information exchange

	*n*	Level 0	Level 1	Level 2	Level 3
Target group focus	62	3 (4.7%)	27 (43.5%)	29 (46.8%)	3 (4.8%)
Development of joint materials	61	11 (18.0%)	20 (32.8%)	20 (32.8%)	10 (16.4%)
Design and corporate identity	62	13 (21.0%)	19 (30.6%)	14 (22.6%)	16 (25.8%)
Dissemination of information/public relations channels	62	6 (9.7%)	30 (48.4%)	21 (33.9%)	5 (8.1%)
Scope and effectiveness of public relations work	62	8 (12.9%)	26 (41.9%)	23 (37.1%)	5 (8.1%)
Cooperation with external bodies	62	3 (4.7%)	6 (9.7%)	34 (54.8%)	19 (30.6%)

### Training and educational programs

Training and educational programs comprised four subdimensions. The subdimension with the highest proportion of RHPCNs at the lowest level (level 0) was “Evaluation of further and advanced education programs,” with 24.2% (*n* = 15) of RHPCNs at this level and further 45.2% of RHPCNs (*n* = 28) at level 1. This subdimension reflected the degree of regular evaluation and the systematic use of results to plan further educational activities. In contrast, the subdimension with the highest proportion of RHPCNs at the highest level (level 3) was “Organization of training and advanced education programs,” with 19.9% (*n* = 12) of RHPCNs achieving this level. This subdimension encompassed the creation of training courses that were regularly accessible to RHPCN partners, often facilitated by permanent training partners or a pool of speakers. Notably, this subdimension also showed the largest concentration of networks at level 1 (33.3%, *n* = 22) and level 2 (39.7%, *n* = 25), indicating that while few networks have reached full maturity in this area, a substantial proportion have progressed beyond the early stages and established intermediate organizational structures for training activities. “Identification of training and advanced education needs” showed the most concentrated distribution, with 61.9% (*n* = 39) of networks at level 1. The “Financing of training and advanced education programs” represented a particular challenge, with over 69.8% of RHPCNs self-assessing in the two lowest maturity levels (level 0: 20.6%, *n* = 13; level 1: 49.2%, *n* = 31). Overall, while some RHPCNs had well-developed structures for training and educational programs, others showed relatively lower levels of maturity in the evaluation, needs assessment, and procurement of sustainable funding for these programs (see Table 7).

**Table 7:** Network maturity regarding training and educational programs

	*n*	Level 0	Level 1	Level 2	Level 3
Identification of training and advanced education needs	63	7 (11.1%)	39 (61.9%)	10 (15.9%)	7 (11.1%)
Organization of training and advanced education programs	61	5 (7.9%)	21 (33.3%)	25 (39.7%)	12 (19.9%)
Financing of training and advanced education programs	61	13 (20.6%)	31 (49.2%)	12 (19.0%)	7 (11.1%)
Evaluation of further and advanced education programs	63	15 (24.2%)	28 (45.2%)	10 (16.1%)	9 (14.5%)

### Development of regional care services and practices

Development of regional care services and practices comprised four subdimensions. The subdimension with the highest proportion of RHPCNs at the lowest level (level 0) was “Development and implementation of new joint projects,” with 21.0% (*n* = 13) of RHPCNs at this level which also showed the largest concentration at level 2 (40.4%, *n* = 21). This subdimension reflected the development and implementation of new healthcare services or care practices, ranging from situational approaches to regular and systematic practices. Conversely, the subdimension with the greatest proportion of RHPCNs at the highest level (level 3) was “Involvement of network members in the development of new joint projects,” with 20.0% (*n* = 10) of RHPCNs achieving this level. This subdimension reflected the active participation of RHPCN members, either through individual efforts, project-related or permanent working groups, or expert groups. Note that this subdimension was collected retrospectively due to a survey display error and is based on a smaller sample (*n* = 52). Both “Development and implementation of new joint projects” and “Evaluation and implementation of new joint projects” showed a pronounced concentration at level 1 (56.5%, *n* = 35 and 57.4%, *n* = 35 respectively). “Identification of joint projects” similarly showed 53.2% (*n* = 33) of networks at level 1, with only 11.3% (*n* = 7) reaching the highest maturity level. 

Overall, the findings indicated that, while some RHPCNs demonstrated highly structured processes for involving members in project development, the identification and evaluation of joint projects was less mature across the sample (see Table 8).

**Table 8:** Network maturity regarding the development of regional care services and practices

	*n*	Level 0	Level 1	Level 2	Level 3
Identification of joint projects	62	4 (6.5%)	33 (53.2%)	18 (29.0%)	7 (11.3%)
Development and implementation of new joint projects	62	13 (21.0%)	35 (56.5%)	8 (12.9%)	6 (9.7%)
Evaluation and implementation of new joint projects	61	7 (11.5%)	35 (57.4%)	12 (19.7%)	7 (11.5%)
Involvement of network members in the development of new joint projects*	52	2(3.8%)	19(36.6%)	21 (40.4%)	10 (19.2%)

*Due to a display error in the online survey, this subdimension was collected retrospectively through a follow-up survey

### Comparison of all subdimensions

Amongst all subdimensions, “Financing“ (dimension: “Infrastructure”) showed the highest proportion of RHPCNs at the lowest level (level 0), with 46.7% (n = 28) of RHPCNs at this level. This suggests that many participating RHPCNs have not yet established structured financial planning and management, including stable funding sources, budgeting, and accounting practices. Similarly, other subdimensions with a high proportion of RHPCNs at the lowest level included: “Evaluation of further and advanced education programs” (dimension: “Training and educational programs”), with 24.2% (n = 15); and “Development and implementation of new joint projects” (dimension: “Development of regional care services and practices”), with 21.0% (n = 13). These figures indicate challenges in the systematic planning and execution of new education programs and healthcare initiatives.

In contrast, the subdimension with the highest proportion of RHPCNs at the highest level (level 3) was “Structure of network meetings” (dimension: “Moderation”), with 42.6% (*n* = 26) of RHPCNs achieving this level. This indicates that many RHPCNs had structured and strategic approaches to the organization of RHPCNs meetings, including practices such as taking and sharing meeting minutes. Other subdimensions with a relatively high proportion of RHPCNs at the highest level included: “Cooperation with external bodies” (dimension: “Public relations and information exchange”), with 30.6% (*n* = 19); and “Enrollment of network members” (dimension: “Establishment and development”), with 30.4% (*n* = 21). This suggests that external collaboration and the structured recruitment of new members were well developed in many RHPCNs.

Overall, findings indicate that participating RHPCNs have made comparatively more progress in formalizing meeting structures, external partnerships, and membership processes, while financial planning, educational evaluation, and the development and implementation of joint projects represent the most substantial areas for further development.

### Comparison of medians between coordination types

For the analysis of subdimension medians across coordination types, data were grouped into four categories: (1) no coordination, (2) only voluntary coordination, (3) at least one paid coordinator employed directly by the network or by network partners, and (4) some type of coordination.

In the “Role of coordination” subdimension, RHPCNs without coordination had a median value of 0, whereas RHPCNs with voluntary, employed, or any form of coordination had a median value of 2, indicating a more structured and established role of coordination, approaching a state of hand.

Similarly, in the “Role of network members” subdimension, RHPCNs without coordination had a median of 0, whereas RHPCNs with voluntary coordination showed a median of 2, and RHPCNs with either employed or any form of coordination had a median of 1, suggesting more clearly defined roles for their members.

The “Definition of topics” subdimension followed a similar pattern, with RHPCNs without coordination exhibiting a median of 0, and all coordinated RHPCNs displaying a median of 1. This indicates that structured coordination was associated with greater involvement in the definition of relevant topics within the RHPCNs.

Regarding the “Structure of network meetings” subdimension, RHPCNs without coordination had a median of 2, while all other coordination types displayed a median of 2. This suggests that structured meetings could still occur even in the absence of formal coordination. However, the “organization of network meetings” varied significantly: RHPCNs without coordination had a median of 0, voluntary coordination RHPCNs had a median of 2, employed coordination RHPCNs showed a median of 1, and RHPCNs with any coordination exhibited a median of 2.

Overall, RHPCNs with employed coordination or any form of structured coordination consistently demonstrated higher maturity levels across subdimensions compared to those with voluntary or no coordination. These findings underline the importance of coordination structures in enhancing the efficiency and development of RHPCNs. In the present study, coordinator background was not analyzed as a stratifying variable, as the maturity model is designed to capture network-level characteristics. The professional training of the individual completing the survey was not assumed to systematically influence the network-level self-assessment. Detailed information on coordinator profiles in German RHPCNs is available in a prior publication [31]. Of the RHPCNs with at least one employed coordinator that provided information on funding of the network (*n* = 34), *n* = 23 (67.6%) reported receiving § 39d funding, while 11 (32.4%) did not (see Table 9).

**Table 9:** Comparison of medians between coordination type

		*N*	No coordination *n* = 7	Only voluntary coordination *n* = 10	At least one employed coordinator* *n* = 46	Some kind of coordination *n* = 56
Moderation	Role of coordination (nature of coordination)	63	0	2	2	2
	Role of network members	63	0	2	1	1
	Definition of topics	63	0	1	1	1
	Structure of network meetings	61	2	2	2	2
	Organization of network meetings	61	0	2	1	2
	Exchange of information	63	1	1	2	2
Establishment and development	Identification and approach of network members	63	2	2	2	2
	Enrollment of network members	63	1	1	1	1
	Needs identification of (potential) network members	63	1	1	1	1
	Objectives	62	1	2	0	2
	Target area: structure/region	63	1	2	2	2
Infrastructure	Communication: data storage and information distribution	62	0	2	2	2
	Financing	60	0	0	1	1
	Legal form of organization	58	1	2	2	2
Public relations and information exchange	Target group focus	62	1	2	2	2
	Development of joint materials	61	0	1	2	2
	Design and corporate identity	62	0	2	2	2
	Dissemination of information/public relations channels	62	1	1	1	1
	Scope and effectiveness of public relations work	62	1	1	2	1
	Cooperation with external bodies	62	1	2	2	2
Training and educational programs	Identification of training and advanced education needs	63	1	1	1	1
	Organization of training and advanced education programs	63	1	2	2	2
	Financing of training and advanced education programs	63	0	1	1	1
	Evaluation of further and advanced education programs	62	1	1	1	1
Development of regional care services and practices	Identification of joint projects	62	1	1	1	1
	Development and implementation of new joint projects	62	0	1	1	1
	Evaluation and implementation of new joint projects	61	0	1	1	1
	Involvement of network members in the development of new joint projects	50	2	1	2	2

* employed by the network directly or by network partners

## Discussion

The primary objective of the present study was to provide a first national assessment of RHPCN development in Germany using a maturity model developed specifically for this context. The analysis revealed strengths and weaknesses across various dimensions, offering valuable insights into the current state of network development. Across 28 subdimensions, self-assessments reveal considerable heterogeneity. The highest maturity scores were observed in the dimensions of Establishment and development, Moderation, and Public relations and information exchange. The dimension of Infrastructure received the weakest self-assessments overall, driven by the subdimension “Financing” (46.7% at Level 0). A factor contributing to the different levels of maturity may be the extent of structured coordination: RHPCNs with employed coordination tended to demonstrate higher levels of maturity in areas such as funding, training, and project development. In the context of networking in end-of-life care, results of further studies of the HOPAN project indicate that paid coordinators are generally more available and able to dedicate a greater number of hours to their responsibilities compared to volunteers [31]. Correspondingly, networks with voluntary or no coordination often demonstrated challenges related to financial stability, systematic training initiatives, and the execution of joint projects, which may be associated with a lack of centralized decision-making and resource management.

Another trend observed was that RHPCNs with structured coordination mechanisms tended to exhibit more advanced knowledge-sharing practices and clearer strategic objectives, often accompanied by designated communication roles and standardized reporting procedures. Conversely, RHPCNs lacking formal coordination seem to struggle more often with ambiguous goal setting and inefficient information flow.

The findings further indicated that coordinated RHPCNs were more likely to support education and training initiatives, with better access to funding, strategic resource allocation, and structured professional development efforts. In contrast, uncoordinated RHPCNs more often lacked the capacity to systematically organize training programs. Similarly, the development and implementation of joint projects was more often more advanced in RHPCNs with structured or even paid coordination.

Overall, the results of the comparison showed that RHPCNs with some type of coordination more often reported a higher level of self-assessed maturity in organizational structure characterized by systematic communication and planning. This observation aligns with the broader literature on coordination, emphasizing the importance of coordination frameworks in providing clear direction and effectively managing complex dynamics within healthcare networks [5, 8, 14, 15, 33, 34].

In addition, in the literature effective coordination has been identified as a key success factor for overall healthcare delivery within networks, particularly through the implementation of standardized practices and centralized capacities [8, 11, 34–36]. These findings further highlight the necessity for robust coordination systems to promote healthcare efficiency and effectiveness.

### Practical implications

The present findings have practical implications for stakeholders involved in the planning, development, and management of RHPCNs. Decision-makers should prioritize the establishment of coordination roles which seem to support a more seamless execution of network activities to enhance network efficiency and sustainability. In contexts where paid coordination is not feasible, stakeholders should determine and train volunteer coordinators in the management of RHPCN activities and moderation of collaboration-building among members.

Moreover, RHPCNs should actively seek to optimize the utilization of available funding. This may involve the formulation of clear strategies to secure and manage financial resources which shows associations with higher network maturity.

Finally, the RHPCNs and their members or coordinators should establish a framework to promote standardized procedures, centralized capacities, and effective leadership, as networks with structured coordination have shown a higher maturity overall. Future initiatives should also aim to establish and nurture partnerships likely to bolster RHPCNs’ capacity to leverage resources and effectively navigate the complexities of healthcare dynamics.

### Strengths and limitations

The present study employed a detailed data collection method to capture diverse aspects of RHPCNs, resulting in a broad first overview of development of German RHPCNs. A further strength of the research lies in its inclusion of participating RHPCNs, representing a wide range of organizational structures from different regions of Germany, at various levels of maturity and different kinds of coordination. The total population of all RHPCNs was unknown at the time of the survey, which precluded the calculation of a response rate. This is a limitation of the study because the potential for non-response cannot be estimated. Further, RHPCNs with more formalized coordination structures may have had more temporal capacities to participate. Conversely, RHPCNs in active developmental processes may be disproportionately represented among respondents, given their likely motivation to engage with a self-assessment instrument. As a result, the findings may reflect the characteristics and self-assessments of participating networks rather than those of German RHPCNs more broadly. Additionally, the challenges identified concerning systematic funding and reliance on project-based resources may be unique to Germany’s healthcare financing model. Consequently, caution should be exercised when extrapolating the present findings to healthcare systems with centralized or privatized funding structures.

The methodological framework of the maturity model inherits both strengths and limitations: primary advantage of the maturity model developed in the present study lies in its capacity to map the development of visions and goals within a practice-oriented framework integrating both qualitative and quantitative approaches, thereby offering considerable flexibility. However, due to the ordinal scaling of the model levels, it was not possible to calculate mean values, thereby limiting the scope of the statistical analysis: the study does not permit causal conclusions. Observed differences in maturity levels across coordination types reflect descriptive patterns and associations only. The maturity model is a self-assessment tool. The findings of the maturity analysis are predicated on the subjective self-assessments of the network coordinators. This study is the first application of this maturity model on a national scale. Further examination of its suitability and consistency across different RHPCNs is needed.

Finally, owing to a display error in the online survey, the subdimension “Involvement of network members in the development of new joint projects” (dimension: “Development of regional care services and practices”) was not correctly displayed. To address this issue, data were collected retrospectively through a follow-up survey, albeit with a smaller number of participants. Despite the reduced sample size, the number of cases remained comparable to those observed for the other subdimensions.

### Implications for future research

Further research is warranted to investigate the observed differences between voluntary and employed coordination, particularly regarding the higher median values in certain subdimensions with respect to voluntary coordination. Exploring factors such as time commitment, intrinsic motivation, interpersonal relationship quality, and perceptions of leadership within the network could provide nuanced insights into these differences. Additionally, it may be important to examine whether such variations reflect broader issues related to the sustainability and long-term impact of coordination within RHPCNs.

Another important avenue for future research is the influence of funding mechanisms on coordination effectiveness. Specifically, financial support provided through § 39d SGB V, warrants closer scrutiny, as this funding mechanism not only enables the compensation of paid coordinators, but it also holds the potential to improve the quality and efficiency of network coordination. The identification of strategies to optimize the use of such funding could significantly enhance network development and long-term sustainability.

Additionally, it would be beneficial to reassess maturity levels at a later stage, once § 39d SGB V funding has been in place for an extended period. Such an analysis would facilitate a more comprehensive understanding of the associations of sustained funding and network maturity over time.

## Conclusions

The present study reflects RHPCNs self-assessed maturity among participating networks in Germany, applying a maturity model developed for this context. Results of the descriptive comparisons point to the relevance of effective coordination and leadership in fostering successful healthcare networks, while also highlighting the need for further validation of the instrument and more rigorous evaluation of RHPCNs in Germany. Future research should incorporate variables such as network size, member types, and external partnerships. Gaining a deeper understanding of the interrelationships between these factors may significantly enhance our comprehension of the factors contributing to RHPCN success, thereby informing the development of strategies aimed at improving performance and sustainability.

## Supplementary Information

Supplementary Material 1.

## Data Availability

The datasets used and analyzed during the present study are available from the corresponding author upon reasonable request.

## Abbreviations

RHPCN: Regional hospice and palliative care network
SAPV: Specialized palliative home care teams
SGB V: Social Security Code, Fifth Book
