# Supplementary material for: State of development of regional hospice and palliative care networks in Germany and a descriptive comparison of the role of coordination: results of an online survey using a maturity model
Source: BMC Palliat Care. 2026 Aug 14;25:250. doi: 10.1186/s12904-026-02279-7 (PMC13480089; doi:10.1186/s12904-026-02279-7)
Supplement: Supplementary file 1 [file 12904_2026_2279_MOESM1_ESM.docx]

**SUPPLEMENTARY MATERIAL: SURVEY QUESTIONNAIRE**

HOPAN Study — Regional Hospice and Palliative Care Networks (RHPCNs) in Germany

**Introduction**

Dear colleagues in hospice and palliative care,

Thank you for your interest in our survey on regional hospice and palliative care networks (RHPCNs) in Germany.

**About the Research Project**

The HOPAN study aims to conduct an inventory and analysis of regional hospice and palliative care networks (RHPCNs) in Germany (regardless of funding under §39d SGB V), to develop tailored recommendations for advancing RHPCN activities based on each network’s stage of development, and to evaluate the usefulness of these recommendations. The project is funded by the Innovation Fund of the Federal Joint Committee (G-BA) (funding code 01VSF22042) and runs from January 2023 to December 2024. For more information about the HOPAN project, please visit the project website.

**About the Maturity Model**

As part of the project, we developed a maturity model for RHPCNs in collaboration with RHPCN coordinators and experts. This model forms the basis for the following survey. The maturity model is designed to help assess a RHPCN’s stage of development across various dimensions, which in turn serves as a foundation for deriving recommendations to support further development.

Ideally, this questionnaire should be completed by someone who is well-acquainted with the structure of the RHPCN. For certain dimensions, however, it may be helpful to involve additional colleagues. You can pause the questionnaire at any time by selecting the relevant option at the bottom left of each page.

**Development of Recommendations**

In the next phase of the HOPAN project, we aim to develop stage-specific recommendations to support the advancement of RHPCNs. We would greatly appreciate it if you share your experiences — both best practices and challenges — in this questionnaire. To ensure you benefit from the resulting recommendations, you may provide your email address at the end of the questionnaire. We will then send you your individual results and tailored recommendations at a later stage of the project.

**Instructions for Completing the Survey**

Completing the survey will take approximately 45–60 minutes, though the time may vary. You may pause the survey and resume it at a later point.

If you are using a mobile device, we recommend completing the survey in landscape mode.

We kindly ask you to participate by January 12, 2024.

If you have any questions regarding the classification within the maturity model, please feel free to contact us by phone or via email (contact details available on each survey page).

**Thank you very much for your participation!**

**Informed Consent**

Ethical research guidelines require that participants in empirical studies give their explicit and informed consent to take part.

**By participating, I confirm that I am of legal age, have understood the purpose of the study, and agree to take part voluntarily. I understand that I can withdraw my consent at any time without providing a reason and without consequences. I also understand that my data will be collected in pseudonymized form and then analyzed and published anonymously.**

- No (do not participate in the survey)
- Yes

**Privacy Policy**

Participation in this study is voluntary.

Your data will be stored and processed in pseudonymized form (meaning that identifying information such as your name and address is replaced by a code—e.g., a reference number—so that identification is only possible with additional information such as a reference list) on the server of the IT center at Hannover Medical School (MHH), in accordance with data protection regulations, particularly the GDPR. The data will be analyzed and published in anonymized form. Temporary pseudonymized storage is necessary to enable the delivery of results. Access to pseudonymized data is restricted to members of the study team only.

Upon completion of the study, your data will be retained for ten years in accordance with good scientific practice guidelines and will then be permanently deleted. You may withdraw from the study at any time without providing a reason. In the event of withdrawal, your data will be deleted unless you explicitly allow its further use in anonymized form for the study.

The consenting individual has the right to withdraw consent at any time with future effect, without providing a reason. From the moment the withdrawal notice is received, your data will be deleted immediately, provided no legal retention requirements apply and the withdrawal is valid. Any data processing conducted prior to the withdrawal remains unaffected.

To withdraw consent, please contact the following email address: hopan@mh-hannover.de

You have the right to obtain information about your stored personal data (Art. 15 GDPR). If you discover that incorrect personal data has been processed, you may request rectification (Art. 16 GDPR).

You also have the right to request the deletion of your personal data under certain conditions. For example, if the data is no longer necessary for the purpose it was originally collected or processed for, or if you withdraw your consent and there is no other legal basis for processing (Art. 17 GDPR). Furthermore, you have the right to restrict the processing of your personal data (Art. 18 GDPR), the right to data portability (Art. 20 GDPR), and a general right to object (Art. 21 GDPR).

If you have any questions or believe your data is being processed unlawfully, you may contact the Data Protection Officer of Hannover Medical School: Datenschutzbeauftrager der MHH, OE 0007, Carl-Neuberg-Strasse 1, 30625 Hannover.

You also have the right to file a complaint with the supervisory authority if you believe your personal data is being processed unlawfully. The responsible supervisory authority for MHH is: Die Landesbeauftragte für den Datenschutz Niedersachsen, Prinzenstraße 5, 30159 Hannover

**Preliminary Questions: Network Characteristics**

In the following section, we would like to ask you a few questions about the RHPCN for which you are completing this questionnaire.

1. **What is the name of the RHPCN?**

The network name will not be included in the analysis or published with the results. It is used solely to check for duplicate entries and for sending individual results.

- *[Open text entry]*

2. **In which federal state is the RHPCN located?**

- Baden-Württemberg
- Bavaria
- Berlin
- Brandenburg
- Bremen
- Hamburg
- Hesse
- Mecklenburg-Western Pomerania
- Lower Saxony
- North Rhine-Westphalia
- Rhineland-Palatinate
- Saarland
- Saxony
- Saxony-Anhalt
- Schleswig-Holstein
- Thuringia

3. **In which district is the RHPCN located?**

- *[Open text entry]*

4. **What area does the RHPCN cover?**

- Parts of the district
- Entire district
- Beyond the district
- Other: *[Open text entry]*

5. **In what year was the RHPCN founded?**

- *[Open text entry]*

6. **How many partners are part of your RHPCN?**

- *[Open text entry]*

7. **Which care providers are involved in your RHPCN? (Multiple selections possible. For example, if multiple specialized palliative home care teams are involved, please select the option only once.)**

- Outpatient home care service
- Inpatient care facility
- Physician
- Hospital
- Outpatient hospice service
- Outpatient children’s hospice service
- Inpatient hospice
- Inpatient children’s hospice
- Specialized palliative home care teams for adults (SAPV)
- Specialized palliative home care teams for children and adolescents
- Advisor for advance care planning (§132g SGB V)
- General municipal or church-based services (e.g. spiritual care, grief counseling)
- Facility for integration assistance
- Care or senior citizens’ support center
- Therapeutic service
- Municipality
- Outpatient cancer counseling center
- Emergency medical service
- Other: *[Open text entry]*

8. **Is your RHPCN structure a provider of specialized palliative home care (SAPV), e.g., with a care contract under §132d para. 1 sentence 6 SGB V?**

- Yes
- No

9. **Is your RHPCN structure an outpatient hospice service, e.g., with funding under §39a para. 2 SGB V?**

- Yes
- No

10. **Is your organization active as an independent regional hospice and palliative care RHPCN, separate from SAPV or outpatient hospice care (with or without funding as a regional hospice and palliative care RHPCN under § 39d SGB V)?**

- Yes
- No

11. **Does your RHPCN pursue commercial interests (e.g. marketing of training programs, promotion of services or products)?**

- Yes
- No

12. **Is your RHPCN content-neutral (e.g. open to providers of both inpatient and outpatient care and to the concerns of all RHPCN partners)?**

- Yes
- No

**The Maturity Model: Explanation and Instructions**

A maturity model is a tool used to classify the progress and development of a particular process or system. It helps to assess how well a particular activity or area is already developed and what steps are needed to improve it further.

It is important to emphasize that a higher or lower level of development does not automatically imply that a network’s characteristics are “good” or “bad”. A higher maturity level means that the process is generally more efficient and stable, while a lower maturity level indicates potential for development. The assessment is relative and depends on the specific objectives and requirements of the network. A low maturity level may be appropriate in certain contexts, while a high maturity level is required in others. It is therefore more a question of measuring the current status and taking appropriate measures, rather than making an absolute assessment of “good” or “bad”.

**Dimensions and subdimensions:** The maturity model comprises six dimensions: (1) Establishment and Development, (2) Training and Educational Programs, (3) Infrastructure, (4) Moderation, (5) Public Relations and Information Exchange, and (6) Development of Regional Care Services and Practices. Each dimension is divided into subdimensions — specific areas within each topic. Definitions for each dimension are provided on the respective pages of the questionnaire.

**Maturity levels:** For each subdimension, you are asked to assign your RHPCN to one of four levels (0–3). Level 0 reflects an early or absent stage of development; level 3 reflects a fully established and systematized practice. If you do not agree with all aspects of a level, please select the level below.

You can pause the survey at any point and continue at a later time. If you have not answered all questions on a page and wish to continue, the system will notify you. If you wish to skip an individual question, click on “I do not want to provide any further information on this page” and click “Continue”. Information already entered on that page will still be saved.

If you have any questions, please call us or send us an e-mail. You can also find the e-mail address on every page by clicking on “Project HOPAN” at the bottom.

**Note:** Throughout this questionnaire, “RHPCN” refers to regional hospice and palliative care network.

**Dimension 1: Establishment and Development**

**Definition: Establishment and development supports network growth through the identification and admission of new members, the analysis of objectives and needs, and the determination of regional network activities and stakeholder engagement.**

Please assign your RHPCN to a level in each subdimension. If you do not agree with all aspects of a level, please select the level below.

13. **Identification and Approach of Network Members**

- **[0]** New RHPCN partners are not sought.
- **[1]** New RHPCN partners are sought by existing RHPCN partners and invited in an unplanned or uncoordinated manner.
- **[2]** New RHPCN partners are actively sought by existing RHPCN partners and the coordination office and invited to join the RHPCN.
- **[3]** New RHPCN partners are actively and systematically sought by existing RHPCN partners and the coordination office (e.g. through identification of gaps in participation) and invited to join the RHPCN.

14. **Enrollment of Network Members**

- **[0]** There is no established procedure for enrolling new RHPCN partners; enrollment is non-transparent and decided by individuals.
- **[1]** There is no established procedure for enrolling new RHPCN partners; however, enrollment is transparent (e.g. RHPCN partners are informed by circular email) and decided by legitimized persons (e.g. coordination, steering group).
- **[2]** There is an established, regular enrollment procedure (e.g. introduction of new partners and voting); enrollment is transparent and decided by legitimized persons.
- **[3]** There is an established, regular enrollment procedure including defined admission criteria (e.g. documented in a cooperation agreement); enrollment is transparent and decided by legitimized persons.

15. **Needs Identification of (Potential) Network Members**

- **[0]** The needs of existing RHPCN partners are not surveyed and are not or only partially known.
- **[1]** The needs of existing RHPCN partners are known from the context of specific situations (e.g. informal discussions).
- **[2]** The needs of existing RHPCN partners are surveyed in a structured manner; the needs of potential RHPCN partners are known from the context of specific situations.
- **[3]** The needs of existing and potential RHPCN partners are surveyed in a structured and regular manner.

16. **Objectives**

- **[0]** The RHPCN does not have common objectives or a network concept.
- **[1]** RHPCN objectives or a network concept are jointly developed and agreed upon within the RHPCN and are known and documented by RHPCN partners (e.g. on the website or in a funding application).
- **[2]** RHPCN objectives or a network concept are jointly developed and agreed upon, known, and documented; they are regularly reviewed.
- **[3]** RHPCN objectives or a network concept are jointly developed and agreed upon, known, documented, and regularly reviewed and updated.

17. **Target Area: Structure/Region**

- **[0]** The target area is not defined (e.g. determined situationally).
- **[1]** The target area is determined by external factors that are assumed as given (e.g. district boundaries).
- **[2]** The target area is selected according to justified criteria and can be flexibly adapted if necessary (e.g. inclusion of services from other regions).
- **[3]** The target area is selected according to justified criteria, regularly reflected upon, and checked or adapted for accuracy and topicality.

**Additional Questions: Establishment and Development**

18. **Is the RHPCN open to all service providers and care facilities, as well as voluntary and community providers in the region?**

- Yes
- No

19. **Does the RHPCN have cooperation agreements with its network members?**

- Yes
- No

20. **What challenges have you experienced regarding establishment and development? (Multiple selections possible)**

- Acquiring new RHPCN partners
- Integration of the RHPCN into existing hospice and palliative care structures
- Lack of understanding of the added value and specific role of the RHPCN
- Other: *[Open text entry]*

21. **Which measures or approaches have proven successful regarding establishment and development?**

- *[Open text entry]*

**Dimension 2: Training and Educational Programs**

**Definition: Training and educational programs enhance members’ qualifications through training and advanced education. This dimension involves the identification of training needs, the organization and funding of courses, and the evaluation of their effectiveness and quality.**

Please assign your RHPCN to a level in each subdimension. If you do not agree with all aspects of a level, please select the level below.

22. **Identification of Training and Advanced Education Needs**

- **[0]** The training and advanced education needs of RHPCN partners are not surveyed and are not or only incompletely known.
- **[1]** The training and advanced education needs of individual RHPCN partners are known only from the context of specific situations (e.g. incidental conversations during meetings).
- **[2]** The training and advanced education needs of all RHPCN partners are known and surveyed in a structured manner.
- **[3]** The training and advanced education needs of all RHPCN partners are known and surveyed in a structured and regular manner (e.g. annually).

23. **Organization of Training and Advanced Education Programs**

- **[0]** No training or advanced education courses are initiated, arranged, or organized.
- **[1]** Training and advanced education courses are made available to RHPCN partners on a situational basis (e.g. when places are available).
- **[2]** Training and advanced education courses are regularly made available to RHPCN partners.
- **[3]** Training and advanced education courses are regularly made available to RHPCN partners; the RHPCN has permanent training partners or a pool of speakers.

24. **Financing of Training and Advanced Education Programs**

- **[0]** There are no funding arrangements for training and advanced education programs.
- **[1]** Funding for training and advanced education courses is arranged on a situational basis.
- **[2]** Established funding models for training and advanced education programs are in place.
- **[3]** Funding models for training and advanced education are established, agreed, and documented within the RHPCN and are regularly adapted to requirements.

25. **Evaluation of Training and Advanced Education Programs**

- **[0]** Training and advanced education courses are not evaluated.
- **[1]** Training and advanced education courses are evaluated on a situational basis.
- **[2]** Training and advanced education courses are regularly evaluated.
- **[3]** Training and advanced education courses are regularly evaluated; results are systematically taken into account when planning new courses.

**Additional Questions: Training and Educational Programs**

26. **What challenges have you experienced regarding training and educational programs? (Multiple selections possible)**

- Training and advanced education needs of RHPCN partners not known
- Insufficient funding for training and advanced education
- Lack of authorization to offer training and advanced education courses in-house
- Difficulty recruiting participants for training and advanced education courses
- Other: *[open text entry]*

27. **Which measures or approaches have proven successful regarding training and educational programs?**

- *[Open text entry]*

**Dimension 3: Infrastructure**

**Definition: Network infrastructure encompasses all measures needed for the physical and organizational operation of the network, including communication structure, financing, IT infrastructure, and legal form. These components serve as the foundation for all network activities.**

Please assign your RHPCN to a level in each subdimension. If you do not agree with all aspects of a level, please select the level below.

28. **Communication: Data Storage and Information Dissemination**

- **[0]** Documents and information (e.g. statutes, concepts, minutes, certificates) are stored by individual RHPCN partners; there is no clear responsibility for data storage and information dissemination.
- **[1]** Documents and information are stored by individual RHPCN partners; a concept for data storage and information dissemination is in progress.
- **[2]** Documents and information are collected and made available on request; clear responsibility for data storage and information dissemination is established.
- **[3]** Documents and information are available in an up-to-date version on a shared communication platform; access for RHPCN partners is transparently regulated; multiple channels are available for information dissemination suited to different target groups.

29. **Financing**

- **[0]** No systematic funding sources; resources are acquired on a project-by-project basis with no or only receipt-based documentation.
- **[1]** Financial resources (e.g. funding or capital contributions) are procured for specific activities; cost statements and project plans are documented.
- **[2]** Systematic and targeted acquisition of financial resources (e.g. membership fees, revenues, public funding); transparent and standardized cost statements are in place.
- **[3]** Systematic and targeted acquisition of financial resources; an annual investment and project plan is prepared as a basis for decision-making by relevant committees; resource management is supported by suitable software (e.g. for accounting).

30. **Legal Form of Organization**

- **[0]** No consideration has been given to the legal form of the RHPCN.
- **[1]** Consideration has been given to the legal form, but no decision has yet been made.
- **[2]** A decision has been made on the legal form that is compatible with the objectives of the RHPCN.
- **[3]** A decision has been made on the legal form that is compatible with the objectives of the RHPCN; this decision is regularly reviewed.

**Additional Questions: Infrastructure**

31. **Does your RHPCN have its own premises?**

- Yes
- No

32. **Does your RHPCN have financial resources or grants?**

- Yes
- No

33. **From which sources does your RHPCN receive funding? (Multiple selections possible)**

- Donations
- Membership fees
- Municipality
- Network coordination funding under §39d SGB V
- Foundation
- Other: *[Open text entry]*

34. **Did your RHPCN receive funding under §39d SGB V in 2023?**

- Yes
- No

35. **Has your RHPCN applied for funding under §39d SGB V for 2024?**

- Yes
- No

36. **What challenges have you experienced regarding infrastructure? (Multiple selections possible)**

- Lack of municipal support for §39d SGB V applications
- Difficulties agreeing on the applicant structure in the region for §39d funding
- Formalities in the application process (e.g. funding only released after a signed employment contract with the coordinator)
- Scope of coordination position funded under §39d SGB V insufficient
- Limited funding period and annual application requirement for §39d SGB V
- Eligibility criteria under §39d SGB V too restrictive
- Understaffing of coordinator
- Insufficient funding for networking activities
- Lack of premises for RHPCN meetings
- Insufficient workplace equipment for the coordinator
- Other: *[Open text entry]*

37. **Which measures or approaches have proven successful regarding infrastructure?**

- *[Open text entry]*

**Dimension 4: Moderation**

**Definition: Network moderation serves a central, neutral, steering role at an operational level, typically carried out by a head office. Depending on the network’s legal form, this role may be assumed by coordinators. Responsibilities include the coordination of network communication and cooperation, needs assessment and agenda setting, the structuring and organization of network meetings, and the management of information and process flows.**

Please assign your RHPCN to a level in each subdimension. If you do not agree with all aspects of a level, please select the level below.

38. **Role of Coordination (Nature of Coordination)**

- **[0]** The role, including tasks and areas of responsibility, is not defined or is unclear; coordination is carried out situationally.
- **[1]** The role, including tasks and areas of responsibility, is fulfilled continuously in day-to-day practice, though not formally defined.
- **[2]** The role, including tasks and areas of responsibility, is agreed between the coordination office and the steering group (e.g. board or small group).
- **[3]** The role, including tasks and areas of responsibility, is agreed with the entire RHPCN and clearly defined, e.g. through joint adoption and documentation.

39. **Role of Network Members**

- **[0]** The role of network members, including tasks and expectations, is not defined or is unclear; roles are fulfilled situationally.
- **[1]** The role of network members, including tasks and expectations, is fulfilled continuously as part of everyday practice, though not formally defined.
- **[2]** The role of network members, including tasks and expectations, is known to all RHPCN partners, e.g. through onboarding.
- **[3]** The role of network members, including tasks and expectations, is governed by binding regulations with a joint commitment of all participants.

40. **Definition of Topics**

- **[0]** Topics are generated from everyday situations or through loose, unstructured collection; topic setting is situational.
- **[1]** Topics are generated in a structured but situational manner, e.g. using tools or defined processes such as collecting agenda items by email ahead of meetings.
- **[2]** Topics are generated in a structured and forward-looking manner using tools or defined processes, e.g. a topic calendar.
- **[3]** Topics are generated in a structured and forward-looking manner; topics are set based on common strategic goals, e.g. with reference to relevant policy developments.

41. **Structure of Network Meetings**

- **[0]** Meetings have a non-binding character with loose topic collection; no minutes are taken.
- **[1]** Meetings include loose topic collection; minutes are taken.
- **[2]** Meetings are prepared (agenda, input gathering, structuring questions); minutes are taken.
- **[3]** Meetings are prepared (agenda, input gathering, structuring questions, prioritization); minutes are taken and comprehensive follow-up is conducted.

42. **Organization of Network Meetings**

- **[0]** Dates, format, and rooms (if applicable) are organized spontaneously; virtual meetings take place without reflection on access requirements.
- **[1]** Dates, format, and rooms are routinely planned; dates are predetermined (e.g. quarterly meetings always in the same room); if virtual meetings take place, always via the same tool.
- **[2]** Dates, format, and rooms are routinely planned; scheduling includes consultation with participants; virtual meetings are organized in accordance with IT security guidelines.
- **[3]** Dates, format, and rooms are routinely planned with early scheduling and participant consultation; virtual meetings are based on coordinated software licenses.

43. **Exchange of Information**

- **[0]** There is no exchange of information between RHPCN partners.
- **[1]** Information is exchanged within the framework of organized meetings.
- **[2]** Information is exchanged within meetings and on a topic-specific basis (e.g. through working groups); information is accessible to all RHPCN partners (e.g. by email or via the homepage).
- **[3]** Information is transparently accessible to all RHPCN partners for participation (e.g. via a shared platform or cloud storage); knowledge sharing is conducted openly and willingly.

**Additional Questions: Moderation**

44. **How often do meetings of all RHPCN members take place?**

- Weekly
- Monthly
- Once per quarter
- Every six months
- Annually
- As required
- Other: *[Open text entry]*

45. **In what form do the meetings currently take place?** Multiple selections possible.

- face-to-face
- virtual (video conference)
- hybrid (streamed face-to-face meetings with the option of virtual participation)

46. **Where do the face-to-face or hybrid meetings of all the RHPCN members currently take place?** Multiple selection possible.

- Premises of the municipality
- Premises of the RHPCN
- Premises of RHPCN members
- Rooms rented externally
- Other: *[Open text entry]*

47. **How are RHPCN members mainly invited to RHPCN meetings?** Multiple selections possible.

- Letter
- E-mail
- Fax
- Telephone
- Chat group
- Homepage
- Other: *[Open text entry]*

48. **Does your RHPCN have one or more coordinators? (Multiple selections possible)**
    *Filter question; if answer is “No,” questions 49–56 are automatically skipped*

- Yes, employed coordination
- Yes, voluntary coordination
- Yes, coordination by RHPCN partners
- No

**Below we have a few questions about your coordinators. Please assign all coordinators in your RHPCN to a number so that we can collate the details of one person. If you have fewer coordinators than listed, you can leave the other answer options blank.**

49. **Gender**

| Coordinator 1 | - Female | - Male | - Other |
| --- | --- | --- | --- |
| Coordinator 2 | - Female | - Male | - Other |
| Coordinator 3 | - Female | - Male | - Other |
| Coordinator 4 | - Female | - Male | - Other |
| Coordinator 5 | - Female | - Male | - Other |
| Coordinator 6 | - Female | - Male | - Other |

50. **Age (in years)**

| Coordinator 1 | - *[Open text entry]* |
| --- | --- |
| Coordinator 2 | - *[Open text entry]* |
| Coordinator 3 | - *[Open text entry]* |
| Coordinator 4 | - *[Open text entry]* |
| Coordinator 5 | - *[Open text entry]* |
| Coordinator 6 | - *[Open text entry]* |

51. **Professional background**

| Coordinator 1 | - Nursing / Nursing Sciences - Education / Social Work - Administration / Economics / Health Management - Social Sciences / Humanities / Public Health - Physiotherapy - Occupational Therapy - Speech and Language Therapy - Medicine - Psychology (incl. curative therapy training) - Other: *[Open text entry]* |
| --- | --- |
| Coordinator 2 | - Nursing / Nursing Sciences - Education / Social Work - Administration / Economics / Health Management - Social Sciences / Humanities / Public Health - Physiotherapy - Occupational Therapy - Speech and Language Therapy - Medicine - Psychology (incl. curative therapy training) - Other: *[Open text entry]* |
| Coordinator 3 | - Nursing / Nursing Sciences - Education / Social Work - Administration / Economics / Health Management - Social Sciences / Humanities / Public Health - Physiotherapy - Occupational Therapy - Speech and Language Therapy - Medicine - Psychology (incl. curative therapy training) - Other: *[Open text entry]* |
| Coordinator 4 | - Nursing / Nursing Sciences - Education / Social Work - Administration / Economics / Health Management - Social Sciences / Humanities / Public Health - Physiotherapy - Occupational Therapy - Speech and Language Therapy - Medicine - Psychology (incl. curative therapy training) - Other: *[Open text entry]* |
| Coordinator 5 | - Nursing / Nursing Sciences - Education / Social Work - Administration / Economics / Health Management - Social Sciences / Humanities / Public Health - Physiotherapy - Occupational Therapy - Speech and Language Therapy - Medicine - Psychology (incl. curative therapy training) - Other: *[Open text entry]* |
| Coordinator 6 | - Nursing / Nursing Sciences - Education / Social Work - Administration / Economics / Health Management - Social Sciences / Humanities / Public Health - Physiotherapy - Occupational Therapy - Speech and Language Therapy - Medicine - Psychology (incl. curative therapy training) - Other: *[Open text entry]* |

52. **Does the coordinator have an (advanced) training in palliative care?**

| Coordinator 1 | - Yes | - No |
| --- | --- | --- |
| Coordinator 2 | - Yes | - No |
| Coordinator 3 | - Yes | - No |
| Coordinator 4 | - Yes | - No |
| Coordinator 5 | - Yes | - No |
| Coordinator 6 | - Yes | - No |

53. **Hours per week spent on coordination** (please estimate for volunteer coordinators)

| Coordinator 1 | - *[Open text entry]* |
| --- | --- |
| Coordinator 2 | - *[Open text entry]* |
| Coordinator 3 | - *[Open text entry]* |
| Coordinator 4 | - *[Open text entry]* |
| Coordinator 5 | - *[Open text entry]* |
| Coordinator 6 | - *[Open text entry]* |

54. **Since when has the coordinator been active in this role?** (MM/YY)

| Coordinator 1 | - *[Open text entry]* |
| --- | --- |
| Coordinator 2 | - *[Open text entry]* |
| Coordinator 3 | - *[Open text entry]* |
| Coordinator 4 | - *[Open text entry]* |
| Coordinator 5 | - *[Open text entry]* |
| Coordinator 6 | - *[Open text entry]* |

55. **Does the coordinator work for the network on a professional or volunteer basis?**

| Coordinator 1 | - Employed by the RHPCN | - As part of the duties associated with working for a network partner | - Volunteering at the RHPCN |
| --- | --- | --- | --- |
| Coordinator 2 | - Employed by the RHPCN | - As part of the duties associated with working for a network partner | - Volunteering at the RHPCN |
| Coordinator 3 | - Employed by the RHPCN | - As part of the duties associated with working for a network partner | - Volunteering at the RHPCN |
| Coordinator 4 | - Employed by the RHPCN | - As part of the duties associated with working for a network partner | - Volunteering at the RHPCN |
| Coordinator 5 | - Employed by the RHPCN | - As part of the duties associated with working for a network partner | - Volunteering at the RHPCN |
| Coordinator 6 | - Employed by the RHPCN | - As part of the duties associated with working for a network partner | - Volunteering at the RHPCN |

56. **Is the coordinator also employed by a RHPCN partner on a separate contract?**

| Coordinator 1 | - Yes | - No |
| --- | --- | --- |
| Coordinator 2 | - Yes | - No |
| Coordinator 3 | - Yes | - No |
| Coordinator 4 | - Yes | - No |
| Coordinator 5 | - Yes | - No |
| Coordinator 6 | - Yes | - No |

57. **What rules or obligations apply to RHPCN partners?** (Multiple selections possible)

- Passing on information after participation in conferences or symposia
- Organization of conferences
- Organization of RHPCN meetings
- Regular participation in RHPCN meetings
- Rotating preparation and distribution of meeting minutes
- Sharing specialist knowledge at RHPCN level (e.g. impulses, presentations)
- Providing support services for those affected
- Transparency and dissemination of information (dates, minutes, handouts, etc.)
- External representation of the RHPCN
- I am not aware of any rules or obligations
- Other: *[Open text entry]*

58. **What challenges have you experienced regarding moderation?** (Multiple selections possible)

- Unclear competencies and responsibilities between coordination and RHPCN partners
- Maintaining neutrality
- Unknown objectives and expectations of RHPCN partners
- Lack of agreement on common goals
- Insufficient involvement of RHPCN partners
- Insufficient commitment from RHPCN partners
- Competition or conflicts between RHPCN partners
- Other: *[Open text entry]*

59. **Which measures or approaches have proven successful regarding moderation?**

- *[*Open *text entry]*

**Dimension 5: Public Relations and Information Exchange**

**Definition: Public relations ensures the network is perceived as credible and established by the public and, above all, by target groups. It involves understanding the target group’s needs, developing information materials, disseminating content, and collaborating with external bodies.**

Please assign your RHPCN to a level in each subdimension. If you do not agree with all aspects of a level, please select the level below.

60. **Target Group Focus**

- **[0]** Target groups and their needs are not identified.
- **[1]** Target groups and their needs are identified; public relations work is not yet tailored to the target groups.
- **[2]** Target groups and their needs are known; public relations work is structured and regularly adapted to the target groups (e.g. different material variants for different audiences).
- **[3]** Target groups and their needs are known; new target groups beyond existing addressees are regularly analyzed; public relations work is structured, regularly adapted, and subject to regular review (e.g. reach analysis).

61. **Development of Joint Materials**

- **[0]** There are no original RHPCN materials.
- **[1]** There are general RHPCN materials (e.g. a network information brochure); RHPCN partners are not involved in their production.
- **[2]** There are general RHPCN materials and topic-specific materials (e.g. on advance care planning or regional care services); RHPCN partners are involved in their production (e.g. to determine needs and provide content).
- **[3]** There are general and topic-specific RHPCN materials; RHPCN partners are actively involved in creating and regularly updating these materials.

62. **Design and Corporate Identity**

- **[0]** There are no RHPCN-specific design elements (e.g. no logo).
- **[1]** There are RHPCN-specific design elements (e.g. a logo), but no standardized design guidelines.
- **[2]** Materials are created in accordance with standardized design guidelines (corporate design); a uniform external presentation is ensured, e.g. through templates.
- **[3]** Materials are created in accordance with corporate design guidelines; a standardized external presentation is ensured through templates; older materials are revised to align with the current corporate design.

63. **Dissemination of Information / Public Relations Channels**

- **[0]** Information is not disseminated.
- **[1]** Information is disseminated via channels established for specific situations (e.g. newsletters via email distribution lists).
- **[2]** Information is disseminated according to an existing concept using target group-specific and content-specific channels (e.g. website, press mailing list, social media).
- **[3]** Information is disseminated according to an existing concept using target group-specific and content-specific channels; the concept is regularly reviewed and updated.

64. **Scope and Effectiveness of Public Relations Work**

- **[0]** Information is not distributed.
- **[1]** Information is made broadly available, e.g. through public displays or website downloads.
- **[2]** Information is actively communicated to the public, e.g. through public information stands or general newsletters.
- **[3]** Information is communicated in a target group-specific and content-specific manner, e.g. through targeted social media campaigns or focus campaigns.

65. **Cooperation with External Bodies** (e.g. regional support centers, information centers, professional associations, higher-level networks)

- **[0]** External bodies are not known and must first be identified.
- **[1]** External bodies are known, but there is no active collaboration.
- **[2]** External bodies are known; cooperation is situational (e.g. use of existing materials on specific topics).
- **[3]** Cooperation with external bodies is regular, structured, and needs-oriented.

**Additional Questions: Public Relations and Information Exchange**

66. **Does your RHPCN have its own website?**

- Yes
- No
- In progress

67. **Does your RHPCN have its own logo?**

- Yes
- No
- In progress

68. **What challenges have you experienced regarding public relations and information exchange?** (Multiple selections possible)

- Lack of awareness about the RHPCN among the general public
- Lack of awareness of the RHPCN among care stakeholders
- Insufficient expertise for public relations work (e.g. layout, social media)
- Other: *[Open text entry]*

69. **Which measures or approaches have proven successful regarding public relations and information exchange?**

- *[Open text entry]*

**Dimension 6: Development of Regional Care Services and Practices**

**Definition: Further development of regional care services and practices aims to improve local care by identifying, developing, implementing, and evaluating joint projects to better meet community needs. Network members are actively engaged throughout this process.**

Please assign your RHPCN to a level in each subdimension. If you do not agree with all aspects of a level, please select the level below.

70. **Identification of Joint Projects (e.g. brochures, regional care standards, information events)**

- **[0]** Ideas for joint projects for the further development of regional care services and practices are not addressed.
- **[1]** Ideas for joint projects (e.g. gaps in care) are collected on a situational basis.
- **[2]** Ideas for joint projects are collected and prioritized in a structured manner.
- **[3]** Ideas for joint projects are collected and prioritized in a structured and regular manner.

71. **Development and Implementation of New Joint Projects**

- **[0]** No joint projects are developed or implemented.
- **[1]** Joint projects are developed and implemented on a situational basis by individuals (e.g. the RHPCN coordinator or an authorized representative).
- **[2]** Joint projects are developed and implemented through project-related or permanent working groups or expert groups, with input from individual RHPCN partners.
- **[3]** Joint projects are developed and implemented through working groups or expert groups and are coordinated and consented with the entire RHPCN (e.g. through structured feedback processes).

72. **Evaluation of New Joint Projects**

- **[0]** Joint projects are not evaluated.
- **[1]** Joint projects are evaluated on a situational basis.
- **[2]** Joint projects are regularly evaluated (e.g. directly after implementation or at regular intervals).
- **[3]** Joint projects are regularly evaluated; results are systematically used to update, revise, or further develop the projects.

73. **Involvement of Network Members in the Development of New Joint Projects**

- **[0]** Due to the absence of joint project development, no network members are involved.
- **[1]** Development is carried out by individuals in isolation (e.g. the RHPCN coordinator or an authorized representative).
- **[2]** Development takes place through project-related or permanent working groups or expert groups, with feedback from individual RHPCN partners.
- **[3]** Development is carried out by working groups or expert groups and is coordinated and consented with the entire RHPCN (e.g. through structured feedback processes).

**Additional Questions: Development of Regional Care Services and Practices**

74. **Have you already developed new regional care services or standards within the RHPCN?**

- Yes, namely: *[Open text entry]*
- No

75. **What challenges have you experienced regarding the development of regional care services and practices?** (Multiple selections possible)

- Different qualifications and quality standards among RHPCN partners
- Lack of willingness among RHPCN partners to agree on common standards or services
- Other: *[Open text entry]*

76. **Which measures or approaches have proven successful regarding the development of regional care services and practices?**

- *[Open text entry]*

**Sociodemographic Questions**

We would now like to ask you a few questions about yourself. Collecting sociodemographic data is standard practice in empirical research and enables a more differentiated analysis of the survey.

77. **What is your gender?**

- Female
- Male
- Other

78. **How old are you? (Please indicate in full years)**

- *[Open text entry]*

79. **What is your highest school-leaving qualification?**

- Finished school without graduation
- Lower secondary school-leaving certificate
- General education school-leaving or certificate or intermediate school-leaving certificate or Intermediate school leaving certificate
- Vocational technical diploma or specialized upper secondary school diploma or higher education entrance qualification (A-levels) or general qualification for university entrance
- Other qualification (e.g. obtained abroad)

80. **What is your highest professional or academic qualification?**

- I am still in training or studying
- I do not have a degree and am not in training or studying
- Apprenticeship or vocational training
- Technical college (master craftsman, technician, or technical academy)
- Bachelor’s degree or intermediate diploma
- Master’s degree, diploma, or equivalent (Examen/Magister)
- Doctorate
- Other: *[Open text entry]*

81. **What is your professional background?** (Multiple selections possible)

- Nursing / Nursing Sciences
- Education / Social Work
- Administration / Economics / Health Management
- Social Sciences / Humanities / Public Health
- Physiotherapy
- Occupational Therapy
- Speech and Language Therapy
- Medicine
- Psychology (incl. curative therapy training)
- Other: *[Open text entry]*

82. **Do you have advanced training in palliative care?**

- Yes
- No

83. **What is your function or role in the RHPCN?**

- Coordination
- Steering group / Board
- RHPCN partner
- Other: *[Open text entry]*

84. **What is your employment type in the RHPCN?**

- Full-time
- Part-time (please specify: *[Open text entry]*)
- Marginal employment
- Voluntary / no paid employment in the RHPCN

85. **How many years have you been working in the RHPCN?**

- *[Open text entry]*

86. **How many years have you been working in the field of hospice and palliative care?**

- Less than 1 year
- 1–3 years
- 3–7 years
- 7–15 years
- More than 15 years

87. **Do you have any other paid employment in addition to your work in the RHPCN?**

- Yes
- No
- I do not have a job directly in the RHPCN

88. **If you have another job: Where are you employed in addition to your work in the RHPCN?**

- Specialized outpatient palliative care team
- Outpatient hospice service
- Inpatient hospice
- Inpatient geriatric care
- Hospital
- Other: *[Open text entry]*

**Further Questions and Data Use**

89. **Upon request, you will receive your individual result of the maturity model assessment and tailored recommendations for your RHPCN’s further development. If you would like to receive these, please enter your email address below. Please note that the mailing will take place at the end of the project.**

- *[Open text entry]*

90. **The German Hospice and Palliative Care Association (DHPV), the German Society for Palliative Medicine (DGP) and the German Medical Association (Bundesärztekammer) are jointly implementing the project ‘Coordination and support of networking efforts in hospice care and palliative care’. The aim over the next few years is to strengthen co-operation between existing RHPCNs and to support establishing new RHPCNs. We are happy to support this project by allowing those responsible at federal and state level to use selected data from our project for their project’s purposes.**

The following information would be forwarded: RHPCN structural data (excluding data from the maturity model)

- No, our RHPCN structure data should not be passed on
- Yes, our RHPCN structure data may be passed on. Please also pass on the following contact details (contact person, telephone number, e-mail address) (voluntary): *[Open text entry]*

91. **We are also happy to support the German Hospice and Palliative Care Guide (www.wegweiser-hospiz-palliativmedizin.de/) with our data.**

The following information would be forwarded: name of the RHPCN, federal state and district, website, size, and §39d SGB V funding status.

- No, our RHPCN data should not be passed on to the guide
- Yes, our RHPCN data may be passed on (contact details voluntary: *[Open text entry]*)

If you are ready to complete the survey, please click ‘Next’ at the bottom right. Please note that you will NOT be able to return if you click on ‘Next’ now. If you would like to change or add information, please use the back button at the bottom left.

**Thank you for your participation.**
